# Expect the unexpected: a case of spontaneous thrombosis of a pial arteriovenous fistula in a preterm newborn with review of the literature

**DOI:** 10.1007/s00381-022-05652-y

**Published:** 2022-09-26

**Authors:** Congedi Sabrina, Moschino Laura, Salvadori Sabrina, Talenti Giacomo, Mainini Nicoletta, Priante Elena, Causin Francesco, Baraldi Eugenio

**Affiliations:** 1 University of Padua, Department of Women’s and Children’s Health, Padova University Hospital, Unit of Neonatal Intensive Care Unit, Division Women’s and Children’s Health , Padua, Italy; 2 Neuroradiology Unit, Padova University Hospital, Padua, Italy; 3 Interventional Neuroradiology, Padova University Hospital, Padua, Italy

**Keywords:** Pial arteriovenous fistula, Preterm, Newborn, Thrombosis

## Abstract

**Introduction:**

Pial arteriovenous fistulas (pAVF) are rare vascular malformations, especially in children and newborns. In neonates, the most common symptom is congestive heart failure.

**Case presentation:**

We report a case of an asymptomatic preterm newborn incidentally diagnosed with pAVF during a routine cranial ultrasound (cUS) on the third day of life. Cerebral magnetic resonance (MRI) confirmed the diagnosis. A wait-and-see approach was chosen by the multidisciplinary team. The cUS and the MRI on day 14 of life showed the spontaneous resolution of the lesion.

**Conclusions:**

This case underlines the challenges in identifying pAVF in the first weeks of life and demonstrates a possible positive outcome for affected neonates.

## Introduction

Neonatal intracranial vascular malformations represent a rare clinical entity. They can be divided into malformations without and with arteriovenous (AV) shunt [1, 2]. The latter comprise different lesions with various etiologies, treatment options, and outcomes, including vein of Galen aneurysmal malformations (VGAMs) and single-hole pial arteriovenous fistulas (pAVFs, also known as non-Galenic fistulas). If VGAMs account for about 1% of the abnormalities of the foetal cerebral arteriovenous system [3, 4], PAVFs are rarely seen in the first year of life, and no extensive epidemiologic data are available. In the largest *available* series of AV malformations published by Lasjaunias et al., neonatal pAVFs accounted for 15% [5]. PAVFs are more frequently localized in the supratentorial compartment [5, 6]. They are defined as abnormal and direct communications between intracranial pial arteries and veins without an interposed capillary bed or malformative nidus. The angio-architectural differences, the age of the patient, and the clinical presentation influence the management strategies [7].

Digital subtraction angiography (DSA) has long been the gold standard to detect and confirm cerebro- and spinovascular lesions, like pAVFs, but has the disadvantages of invasiveness, need for procedural preparation, and expenses. Cerebral magnetic resonance imaging (MRI) with MR angiography can be another, less demanding technique to define pAVFs’ features (origin, size, localization, and angioarchitecture) [8–10].

Clinically, the most common presentation in children is congestive heart failure (from fistula overload) [5, 9], and haemorrhage, among others [10]. Treatment options in newborns are similar to those in the adult population, although limitations exist due to body weight, size of the lesion, and potential comorbidities [7]: endovascular treatment is the preferred approach [11–13].

We describe a unique case of a pAVF incidentally *diagnosed* in an asymptomatic preterm newborn from a neurological point of view showing a benign evolution and no need for surgical or endovascular intervention. A literature review follows this case report.

## Case report

### Perinatal history

A female preterm infant was born via urgent cesarean section at 31^+5^ gestational weeks (GW) due to alteration of the cardiotocographic trace. The pregnancy was characterized by maternal hypertension and intrauterine growth restriction (IUGR) at 30^+6^ GW. No maternal infectious disease markers were present: serologies, blood tests (c-reactive protein, blood count and biochemical profile), and urine culture were negative.

At birth, the newborn was in good condition with normal vital parameters sign*.* At 8 min of life, due to dyspnea, continuous positive airway pressure (CPAP) was started with neonatal resuscitation T-piece. Arterial cordonal blood gas analysis was balanced. Given the gestational age and the need for respiratory support, the newborn was transported to the neonatal intensive care unit (NICU) for further care.

### Neonatal course and investigations

On admission at our NICU (Padova University Hospital, Italy), the baby was alert with a mild hypotonia (explainable by the gestational age and the IUGR). Her birth weight was 1540 gr (40th%ile)*.* Due to increased dyspnoea, a nasal intermittent mandatory ventilation was preferred. An umbilical central venous catheter was placed.

**Fig. 1:**
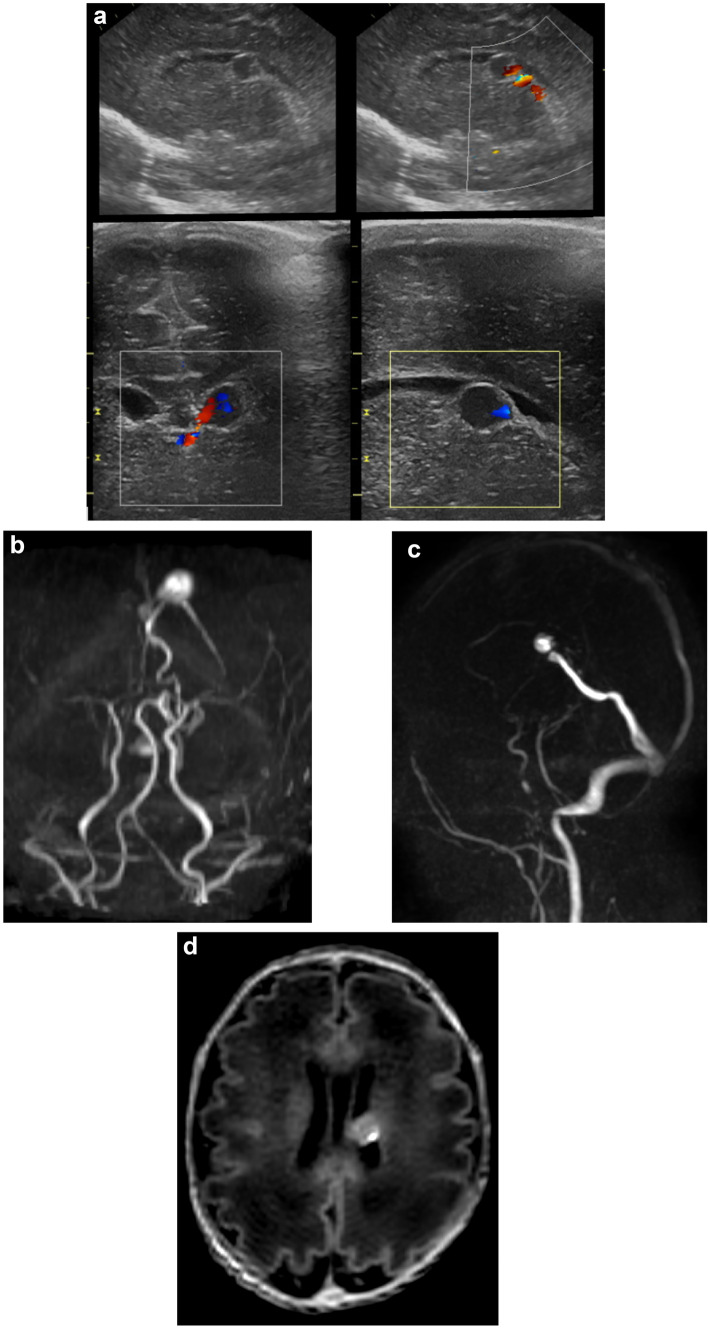
**a** Cerebral ultrasound demonstrating a “cystic” lesion in the left germinal matrix with flow on color Doppler mode. **b** A ToF MR angiography in the arterial phase shows arterial flow in the pouch and the presence of a fistula with two feeders from the posterior cerebral arteries. **c** A single deep venous drainage into the vein of Galen is noted. **d** A T1-weighted MR image demonstrates hyperintensity of the posterior wall of the pouch due to partial thrombosis.

On admission, a cerebral ultrasound (cUS) was performed showing a cystic lesion in the left germinal matrix. However, the following cUS on day 3 of life (DOL) depicted the presence of flow within the cyst on colour Doppler mode, revealing a vascular nature of the lesion (Fig. 1a). Neurological examination was normal. A brain MRI scan was therefore carried out, which demonstrated a flow void in the site of the lesion depicted at cUS, interpreted as a venous pouch. A time-of-flight (ToF) MR angiography showed arterial flow in the pouch due to the presence of a fistula with two feeders from the posterior cerebral arteries and a single deep venous drainage into the vein of Galen. In addition, T1-weighted images demonstrated hyperintensity in the posterior wall of the pouch, due to initial thrombosis (Fig. 1b–d). Considering the radiological features of the lesion, the gestational age, and the weight of the patient, decision was taken for a conservative approach consisting of cardiovascular monitoring and serial cUS examinations (every 2 days). Lastly, an amplitude-integrated-EEG was carried out and resulted to be normal. No other vascular malformations were detected at abdominal US. No genetic investigation was required, given the sporadic nature of the lesion.

### Evolution

**Fig. 2:**
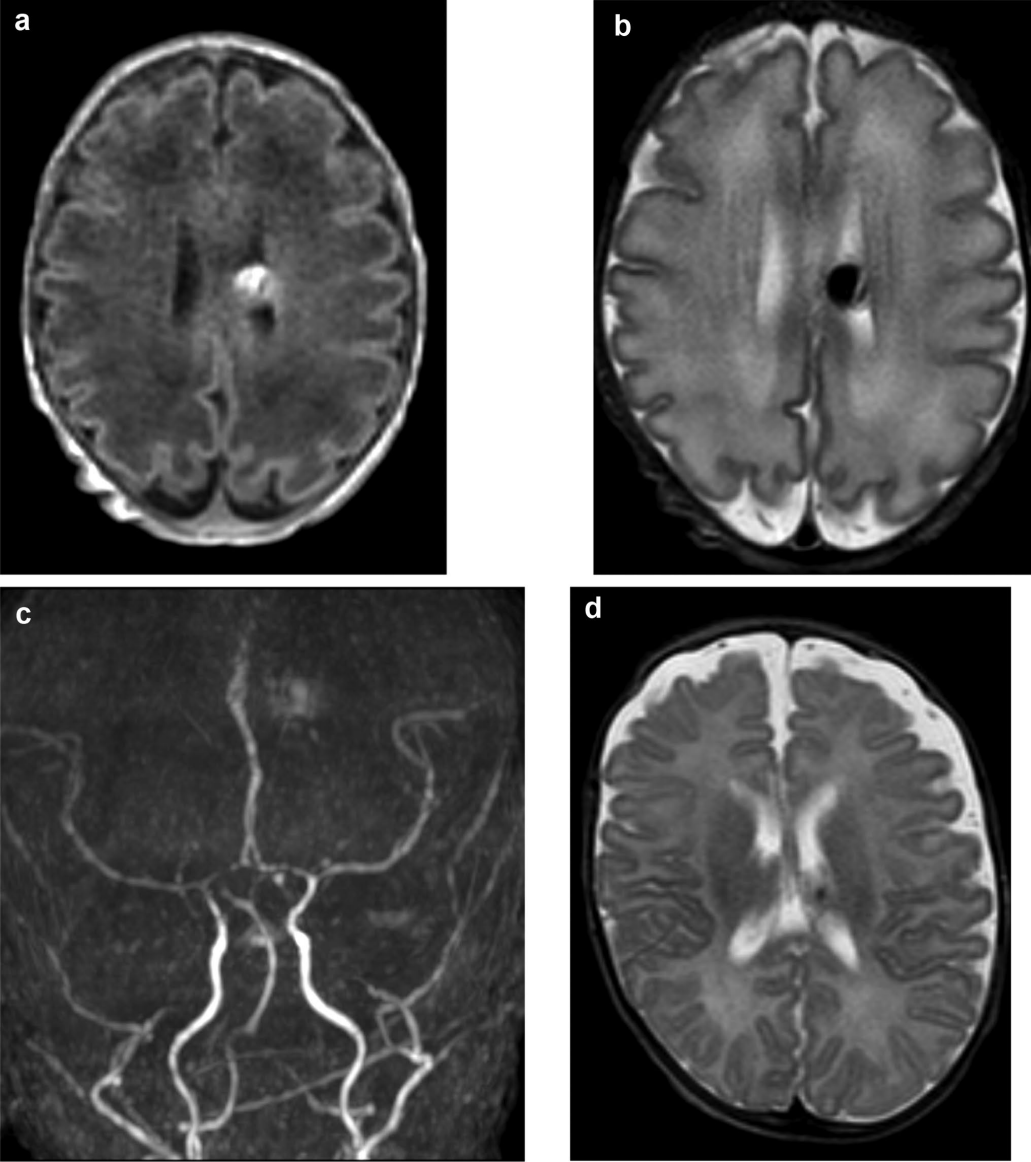
Follow-up brain MR scans: **a**, **b** T1- and T2-weighted images at 14 days showing complete thrombosis of the pouch. **c** A time-of-flight MR angiography shows disappearance of the vascular flow in the feeding vessels. **d** A further follow-up scan after 2 months demonstrates shrinking of the pouch

The patient remained clinically stable for the course of the NICU hospitalization, with normal haematocrit levels. The cUS performed at 14 DOL showed increased echogenicity inside the vascular pouch (Fig. 2a–c). A follow-up brain MRI scan showed disappearance of the vascular flow inside the pouch due to complete spontaneous thrombosis. Additionally, arterial flow was no longer visible in both feeding and draining vessels (Fig. 2c, d).

The baby was discharged at 40^+2^ GW (60th DOL). Her neurological examination was adequate for age with normal head circumference.

The 12-month follow-up visit demonstrated a normal neurological exam with good and appropriate psychomotor evolution for the corrected GA.

An MRI with MR angiography at the same age confirmed the absence of the vascular lesion and its afferent and efferent branches and the remnant small malacic area adjacent to the left lateral ventricle.

## Review of literature

A comprehensive literature search on intracranial arteriovenous fistula in neonates was performed on PubMed/MEDLINE online databases. The following key words and Medical Subject Heading terms were used: “newborn”, “infant”, “neonate”, “pediatric”, “intracranial”, “cerebral”, and “pial arteriovenous fistula”. Articles written in English and published between 2005 and October 2021 were included. Nine articles were selected after the application of the above-listed criteria (Table 1).

**Table 1:** Case reports and case series of review of literature about pial arteriovenous fistula in newborns and infants

**Study**	**Year of publication**	**No. of patients**	**Sex**	**Age**	**Clinical presentation**	**IA location**	**Diagnosis**	**Haemorrhage**	**Treatment modalities**	**Outcomes**
**Maejima et al.** [9]	2018	1	M	At birth	Congestive heart failure, respiratory distress	Right PAVF	MRI, angiography	X	Endovascular treatment	Right cerebral hemisphere atrophy, heart failure, respiratory insufficiency, and finally death
**Pedicelli et al.** [4]	2017	1	M	At birth	No one (prenatal detection of a cerebral lesion)	Right frontal PAVF	MRI, angiography	X	Endovascular treatment, surgery	Normal development
**Martınez-Payo et al.** [14]	2017	1	M	Pre-natal onset	Cerebral ventriculomegaly, cardiomegaly	High-flow PAVF, connected to the left middle cerebral artery	Foetal ultrasound, MRI	X	No one	Spontaneous intrauterine foetal death occurred at 30 weeks of pregnancy
**Kraneburg et al.** [10]	2014	1	M	36 days old	Inconsolable crying, episodes of stiffening of limbs and arching of the neck, shock	PAVF supplied by a branch of the right MCA with a large aneurysm	Head CT, MRI	IVH with hydrocephalus	Endovascular treatment	Normal development
**Cooke et al.** [13]	2012	1	F	9 months	Developmental delay, heart failure at presentation; later, vomiting, loss of consciousness	PAVF in a dilated right basal vein of Rosenthal, supplied by posterior temporal choroidal branches of the posterior cerebral and middle artery	Angiography	IVH with hydrocephalus	Endovascular treatment	Normal development
**Zuccaro et al.** [3]	2010	2	F/M	6 months/10 months	Hydrocephalus, developmental delay, cutaneous angioma/lower limb asymmetry, cutaneous angioma	Left frontal PAVF/ D6—sacrum PAVF	Head CT, MRI/MRI	X	Surgical resection/ endovascular treatment	Developmental delay, shunt/complete recovery
**Zareen et al.** [8]	2010	1	F	3 months	No one	left choroidal artery and dilated basal vein	Head CT, MRI	X	Endovascular treatment	Normal development
**Potter et al.** [15]	2009	1	M	At birth	Respiratory distress, heart failure	Holohemispheric pial arteriovenous malformations	Head CT	X	Endovascular treatment	Partial recovery with left hemiparesis
**Weon et al.** [16]	2004	26	19 M/7F	From birth to 12 months	Heart failure (15), macrocrania (5), epilepsy (5), asymptomatic (1)	Supratentorial localization, no more details	Head CT, MRI	ICH (4), SAH (1)	Endovascular treatment (23)/ no treatment (3)	Neurological defects (2), developmental delay (2)*, (data are uncomplete)*

*M* male, *F* female, *CT* computed tomography, *MRI* cerebral magnetic resonance

Table 1 summarizes data on pAVFS in the neonatal population: 35 patients with diagnosis of pAVFs have been reported in the last 15 years in the neonatal *and infant* population (Table 1). The postnatal incidental detection, the prematurity of the patient, the diagnostic process, the management, and the unusual clinical progression warrant the relevance of reporting our case.

To our knowledge, this is the first case describing an incidental diagnosis and a spontaneous resolution of a pAVF. Moreover, pAVF involving the posterior circulation are rare in newborns and even rarer in preterm newborns [7].

According to our review of the literature, heart failure, epilepsy, and macrocrania are the most common manifestations of pAVFs [3, 4, 7–9, 12–15]. Luckily, our case was not diagnosed due to such clinical presentation but during a routine cUS. This also highlights the role of a non-invasive tool like cUS and the importance of using colour Doppler to evaluate the nature of cystic lesions. cUS may also be useful in the monitoring of these lesions over time. After this case, we have well-defined redefined o reviewed our local protocol, recommending the use of colour Doppler to investigate any cerebral cystic lesions. Post-natal MRI and angiography can be useful, fast, and non-invasive tools to confirm the presence of cerebrovascular lesions and pAVFs. Indeed, almost all cases of arterial aneurysms described in the literature so far were diagnosed or confirmed by MRI and/or angiography after an antenatal suspicion or after a typical clinical presentation [3, 4, 8–10, 13–16].

DSA in newborns should be performed mainly in the case of a life-threatening condition to address a complete or partial occlusion of the AV shunt. The possibility of obtaining detailed diagnostic information on the vascular lesion and the surrounding tissues by means of DSA was rejected in our case in consideration of the low weight of the baby that allowed only a very small amount of contrast to be used. Additionally, the steal phenomenon caused by the AV shunt often prevents a detailed assessment.

Both surgical and endovascular approaches can be used to control the AV shunt. In recent years, thanks to technological improvements and the growing experience of neuroradiologists, endovascular techniques have gained a more important role in treating AV shunts, even in children and newborns [16]. Therefore, the risks and benefits of both endovascular and surgical approaches must be carefully considered, and a case-by-case evaluation should be adopted.

The most relevant feature of our case was the occurrence of a spontaneous thrombosis of the lesion with a good outcome despite the early onset. This favourable course of the disease was the ideal one among the 3 hypothesized during the multidisciplinary meetings. The other possible outcomes were, in fact, the progressive growth of the lesion and the haemorrhagic rupture of the venous pouch. The “wait-and-see” approach was weighed against the option of an endovascular approach, and the decision was guided by the age of the patient and the absence of clinical symptoms related to the lesion. These benefits were considered to be greater than the risk of the procedure.

The clinical stability of the patient radically changed the management plan, with no further need for invasive procedures. So far, no clear explanation for this phenomenon could be proposed.

Given the rarity of the condition, an international database of newborns affected by AV shunts in the first 28 days of life could help in delineating a standardized management of these vascular malformations according to GW and weight. A multidisciplinary operational framework is suggested, which needs to take into account the chance of a positive outcome without any treatment.

## Conclusion

Intracranial vascular malformations are rare conditions in the neonatal population. Few data in preterm infants and no standardized management and therapeutic protocols are available. This case of unexpected spontaneous thrombosis of a cerebral pial arteriovenous fistula in a preterm neonate represents an unusual event. An international collaboration to collect data of affected patients may be useful in the further development of shared guidelines.

## Data Availability

Not applicable.

## Abbreviations

PAVF or pAVF: Pial arteriovenous fistulas
cUS: Cranial ultrasound
MRI: Cerebral magnetic resonance
AV: Arteriovenous
VGAMs: Vein of Galen aneurysmal malformations
GW: Gestational weeks
IUGR: Intrauterine growth restriction
CPAP: Continuous positive airway pressure
NICU: Neonatal intensive care unit
DOL: Day of life
DSA: Digital subtraction angiography
